# [^18^F]FDG and [^18^F]FLT PET for the evaluation of response to neo-adjuvant chemotherapy in a model of triple negative breast cancer

**DOI:** 10.1371/journal.pone.0197754

**Published:** 2018-05-23

**Authors:** Isabella Raccagni, Sara Belloli, Silvia Valtorta, Alessandro Stefano, Luca Presotto, Claudio Pascali, Anna Bogni, Monica Tortoreto, Nadia Zaffaroni, Maria Grazia Daidone, Giorgio Russo, Emilio Bombardieri, Rosa Maria Moresco

**Affiliations:** 1 Institute of Molecular Bioimaging and Physiology (IBFM), CNR, Segrate, Italy; 2 Tecnomed, Foundation of the University of Milano-Bicocca, Monza, Italy; 3 Experimental Imaging Center, IRCCS San Raffaele Scientific Institute, Milan, Italy; 4 Medicine and Surgery Department, University of Milano-Bicocca, Monza, Italy; 5 Nuclear Medicine Unit, IRCCS San Raffaele Scientific Institute, Milano, Italy; 6 Nuclear Medicine Unit, Fondazione IRCCS Istituto Nazionale dei Tumori, Milan, Italy; 7 Molecular Pharmacology Unit, Experimental Oncology and Molecular Medicine Department, Fondazione IRCCS Istituto Nazionale dei Tumori, Milan, Italy; 8 Biomarkers Unit, Experimental Oncology and Molecular Medicine Department, Fondazione IRCCS Istituto Nazionale dei Tumori, Milan, Italy; 9 Nuclear Medicine Department, Humanitas Gavazzeni, Bergamo, Italy; Wayne State University, UNITED STATES

## Abstract

**Rationale:**

Pathological response to neo-adjuvant chemotherapy (NAC) represents a commonly used predictor of survival in triple negative breast cancer (TNBC) and the need to identify markers that predict response to NAC is constantly increasing. Aim of this study was to evaluate the potential usefulness of PET imaging with [^18^F]FDG and [^18^F]FLT for the discrimination of TNBC responders to Paclitaxel (PTX) therapy compared to the response assessed by an adapted Response Evaluation Criteria In Solid Tumors (RECIST) criteria based on tumor volume (Tumor Volume Response).

**Methods:**

Nu/nu mice bearing TNBC lesions of different size were evaluated with [^18^F]FDG and [^18^F]FLT PET before and after PTX treatment. SUV_max_, Metabolic Tumor Volume (MTV) and Total Lesion Glycolysis (TLG) and Proliferation (TLP) were assessed using a graph-based random walk algorithm.

**Results:**

We found that in our TNBC model the variation of [^18^F]FDG and [^18^F]FLT SUV_max_ similarly defined tumor response to therapy and that SUV_max_ variation represented the most accurate parameter. Response evaluation using Tumor Volume Response (TVR) showed that the effectiveness of NAC with PTX was completely independent from lesions size at baseline.

**Conclusions:**

Our study provided interesting results in terms of sensitivity and specificity of PET in TNBC, revealing the similar performances of [^18^F]FDG and [^18^F]FLT in the identification of responders to Paclitaxel.

## Introduction

Breast cancer (BC) is a heterogeneous disease composed of several biological subtypes having different clinical course, response to therapy and molecular profile. The lack of expression of Estrogen Receptor (ER), Progesterone Receptor (PR), Epidermal Growth Factor Receptor 2 (HER2) and the absence of HER2 amplification define the TNBC [1]. TNBC represents approximately 15–20% of all invasive breast cancers and is characterized by ductal histology, high mitotic rates and earlier lymph node involvement when compared to other BC subtypes [2]. TNBC is frequently associated to high expression of proliferation markers as Ki67 and cyclins and activation of the beta-catenin pathway [3].

High aggressiveness, as well as non-susceptibility to hormone and targeted therapies, limits the number of therapeutic opportunities and makes the prognosis of TNBC patients poor. NAC with anthracyclines and the mitotic inhibitors taxanes used in sequential or combined treatment, represents the standard pharmaceutical approach for TNBC [4,5,6] and describes therapeutic interventions prior to surgery to reduce size of unresectable tumors and test therapies efficacy. Despite its intrinsic aggressiveness, TNBC is highly responsive to NAC, a phenomenon called “triple negative paradox” [4,6]. Unfortunately, those patients who do not achieve pathological complete response (pCR) present a high rate of relapse. Therefore, much research is focused on the development of biomarkers predictive of clinical response, avoiding the use of ineffective protocols and customizing the optimal strategy. Traditionally, treatment response has been assessed through the application of RECIST, which classifies effectiveness on the basis of tumor shrinkage, using anatomical measurements. However, this parameter represents a later event compared to other changes which may be triggered by treatments [7]. PET allows the non-invasive monitoring of biological aspects related to tumor growth and aggressiveness, like glucose metabolism, cell proliferation and hypoxia [8]. In different types of cancer, the radioligand 2-deoxy-2-[^18^F]fluoro-D-glucose ([^18^F]FDG) has been reported as useful tool for early prediction of response or resistance to pharmacological treatment [9]. Considering TNBC, a reduction of [^18^F]FDG uptake after two cycles of neo-adjuvant chemotherapy has been recently proposed as a powerful marker of patients’ outcome [10,11,12], but preclinical as well as clinical studies identified other tracers of potential interest. Among these, the thymidine analogue 3’-[^18^F]fluoro-3’-deoxythymidine ([^18^F]FLT) seems to be a potential indicator of tumor response/resistance to therapy [13,14,15]. In fact, the uptake of [^18^F]FLT reflects the activity of the enzyme thymidine kinase-1 (TK1), well known for its function in the pyrimidine salvage pathway. This enzyme is upregulated during late G1/S phase of the cell cycle, thus representing an indirect marker of cell proliferation.

The high basal [^18^F]FDG uptake and rate of cell proliferation make TNBC an adequate subtype of BC to investigate response assessment with PET. Many studies have been performed to compare the effect of repeated chemotherapy on [^18^F]FLT and [^18^F]FDG uptake [16,17,18,19,20,21,22], but data on TNBC are not conclusive. In this study, we aimed to evaluate and compare the effect of NAC with taxane on [^18^F]FDG and [^18^F]FLT uptake in a xenograft model obtained through the subcutaneous injection of human TNBC cells. Moreover, in a small group of mice, we explored the ability of [^18^F]FDG and [^18^F]FLT to predict tumor response to PTX in comparison to objective response evaluation made by Tumor Volume Response (TVR) evaluation at the end of treatment.

## Materials and methods

### Cell culture

MDA-MB-468 cells (ATCC, LGC Standards S.r.l., Italy) were routinely cultured in at 37°C in a 5% CO_2_-humidified incubator using Dulbecco Modified Eagle Medium (DMEM, Sigma Aldrich S.r.l., Italy) supplemented with 10% heat-inactivated fetal calf serum (EuroClone S.p.A., Italy), 2 mM L-glutamine, 1 mM sodium pyruvate, 100 units/ml penicillin and 100 μg/ml streptomycin (EuroClone S.p.A., Italy).

### Animal experiments

All animal experiments were carried out in compliance with the institutional guidelines for the care and use of experimental animals, which have been notified to the Italian Ministry of Health and approved by the ethics committee of the San Raffaele Scientific Institute. Female SCID Hairless Congenic (SHC™) mice (Charles River, Italy) of 6–8 weeks of age were subcutaneously implanted on the back with 1.5 x 10^7^ (n = 24) or 2 x 10^7^ (n = 14) MDA-MB-468 cells under ketamine/xylazine anaesthesia (i.p., 100 mg/kg / 10 mg/kg). Animals were housed in the animal facility of San Raffaele Scientific Institute and daily monitored for body weight and lesions sprouting; tumor volume was measured with digital calliper twice a week and expressed as *(L x l*^*2*^*)/2 = (mm*^*3*^*)* where *L* is the long side and *l* is the short side. Moreover, when tumours reached diameters of more than 15 mm or when mice showed signs of severe illness, they were euthanized by cervical dislocation under isoflurane anaesthesia.

### Treatment protocol

PTX was prepared dissolving the drug powder in the vehicle solution: 90% saline, 5% ethanol and 5% Cremophor (Sigma Aldrich S.r.l., Italy). Tumors smaller than 150 mm^3^ (small tumors, n = 12) or larger than 150 mm^3^ (large tumors, n = 14) were randomized into two groups and treatment started with vehicle (control) or Paclitaxel (treated, 18 mg/kg i.v., two doses per week) for two weeks. Treatment response was evaluated using [^18^F]FDG and [^18^F]FLTPET scans, before (baseline) and at the end of treatment. The efficacy was determined according to the RECIST score adapted to the experimental procedure [23]. Indeed, since the standard monitoring of tumor in preclinical setting is usually performed by volume measurement, an adapted RECIST score was used in the study. This index was defined as Tumor Volume Response (TVR) and calculated as the percentage change in median tumor volume measured by calliper at the end of treatment over the median tumor volume before treatment. According to this definition, treatment response was calculated as Partial Response (PR) (TVR, score at least > -30%); Stable Disease (SD), (TVR, score < -30% and < +20%) and Progressive Disease (PD), (TVR score > +20%) [24].

### PET evaluation

[^18^F]FDG, prepared for clinical use (European Pharmacopeia VIII Edition), and [^18^F]FLT [25] were injected with a radiochemical purity > 99%. PET acquisitions were performed as previously described [13]. Identification of hypermetabolic or hyperproliferative lesions was performed using a segmentation method [26], adapted for preclinical use. Briefly, an algorithm based on Random Walks (RW) on graphs has been used to convert DICOM (Digital Imaging and Communications in Medicine) images into a graph where some nodes are known (nodes with target or background label) and others unknown. PET image is then converted in a lattice where voxel SUVs are assigned to corresponding graph nodes and edge weights are computed accordingly. A probability map is then produced, and a threshold *p* is chosen to discriminate between target and background voxels. Tracers’ uptake was expressed as:

- standardized uptake value (SUV = [radioactivity in the tumor/injected radioactivity]*animal weight);
- metabolic tumor volume (MTV = volume (mm^3^ of the VOI after segmentation);
- total lesion glycolysis (TLG) for [^18^F]FDG or total lesion proliferation (TLP) for [^18^F]FLT = SUV_mean_*MTV.

Variations in all parameters in sequential scans were normalized to baseline and expressed as percentage of variation (% change) according to the following formula:

%change=100x(post‑treatment–pre‑treatment)/pre‑treatment.

### Histological and immunohistochemical analyses

Twelve of the twenty-four female SCID mice implanted with 1.5 x 10^7^ MDA-MB-468 cells were treated with PTX (n = 6) or vehicle (n = 6) were sacrificed for histological (H&E) and immunohistochemical (IHC) analyses for Ki67, as already described [27]. Proliferation index (P.I.) was evaluated for each tumor considering the whole number of Ki67 positive nuclei over the whole number of cell nuclei in three randomly selected fields.

### Statistical analysis

Data generated were expressed as percentage change between the end and the baseline of treatment, mean value with standard deviation (mean±S.D.). Prism 4 (GraphPad Software Inc., San Diego, CA, USA) was used for the statistical analysis. Parameters of radiotracer uptake were assessed and compared through the Student T-test or the ANOVA test using Bonferroni’s multiple comparison; *p* was considered statistically significant, when < 0.05. The accuracy of PET parameters was evaluated by carrying out the Receiver Operating Characteristic (ROC) analysis in defining the pathological response.

## Results

### Tumor weight after treatment correlates with Ki67 expression

**Fig 1:**
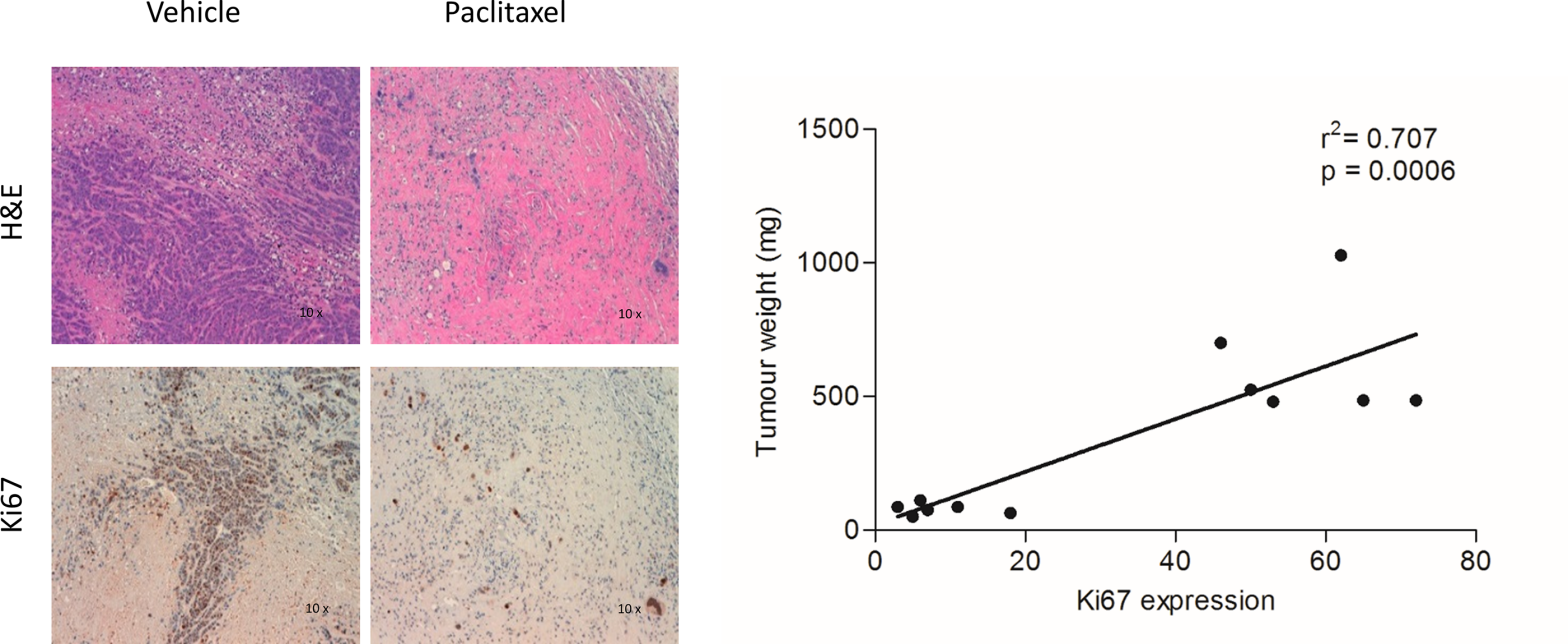
Histological and Ki67 immunohistochemical staining of tumors treated with PTX or vehicle. A) Representative images of histological morphology (H&E) and Ki67 staining of tumors receiving vehicle or PTX. B) Weights of tumors collected at the end of treatment significantly correlated with Ki67 P.I. values (r^2^ = 0.707, p = 0.0006).

We firstly evaluated in a separate group of mice bearing MDA-MB-468 cells the effect of Paclitaxel on Ki67 proliferation marker which is used in clinical practice to assess neo-adjuvant chemotherapy [28]. No animal died because of the experimental procedures or showed signs of illness during tumor growth. The results clearly indicate a reduction of Ki67 staining as a consequence of PTX treatment. Moreover, the weight of harvested tumors (mg) significantly correlated with the corresponding Ki67 expression level (Fig 1).

### Response of MDA-MB-468 tumors to PTX was independent from the initial size

**Fig 2:**
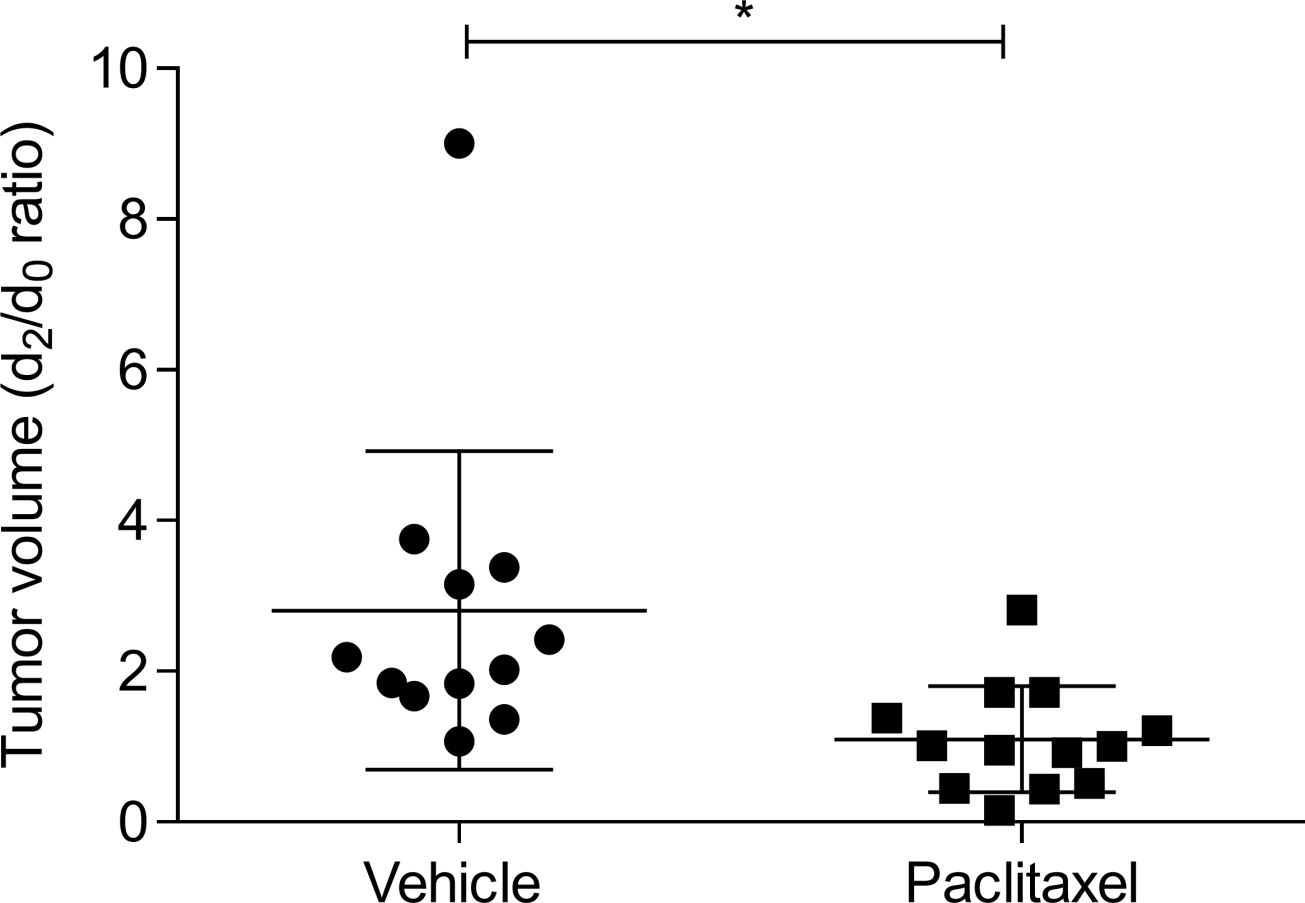
Effect of PTX on MDA-MB-468 tumors. Tumor volume of xenograft mice treated with PTX (4 doses, twice a week, 18 mg/kg i.v.) or vehicle expressed as ratio between post-therapy and baseline. Student’s T test; *p<0,05.

To better represent tumor variability and to mimic the heterogeneity of the human disease, mice which underwent PET evaluations were inoculated with different concentrations of MDA-MB-468 cells and treatment started when tumors reached a volume smaller than 150 mm^3^ (76.7 ± 35.7 small tumors, n = 12), or more than 150 mm^3^ (236.8 ± 107.5 large tumors, n = 14). After the whole PTX cycle, treated animals displayed a significant decrease in tumor volume, when compared to animals receiving vehicle (p = 0.018) (Fig 2). In addition, the response to treatment resulted independent from tumor size at the beginning of treatment. Indeed, applying the TVR for the evaluation of response to PTX therapy, a PR was observed in 33% of small tumors and in 29% of mice bearing large tumors. Similarly, 33% and 43% of mice bearing small and large tumors respectively exhibited SD. Finally, a comparable number of mice bearing small (33%) or large tumors (29%) showed an increase in lesions volume being defined as PD (Table 1), indicating that MDA-MB-468 tumors response to PTX is independent from the initial lesion size.

**Table 1:** Summary of tumor size of treated animals at baseline (pre) and at the end of PTX treatment (post) and the corresponding Tumor Volume Response (TVR) categorization.

Small Tumors (< 150 mm^3^)				Large tumors (> 150 mm^3^)			
	Pre	Post	%TVR		Pre	Post	%TVR
Mouse 1	62.5	32.0	-48.8 (PR)	Mouse 7	162.0	70.0	-56.3 (PR)
Mouse 2	40.0	18.0	-55.0 (PR)	Mouse 8	386.0	60.8	-84.3 (PR)
Mouse 3	75.0	75.0	0.0 (SD)	Mouse 9	575.0	550	-4.3 (SD)
Mouse 4	87.5	105.9	21.0 (SD)	Mouse 10	169.0	171.5	1.5 (SD)
Mouse 5	75.0	211.0	181.3 (PD)	Mouse 11	245.0	225.0	-8.2 (SD)
Mouse 6	135.0	232.8	72.4 (PD)	Mouse 12	208.3	288	38.3 (PD)
				Mouse 13	180.0	309.4	71.9 (PD)

PR = partial responder; SD = stable disease; PD = progressive disease.

### Δ[^18^F]FDG and Δ[^18^F]FLT SUV_max_ are similarly influenced by PTX treatment

**Fig 3:**
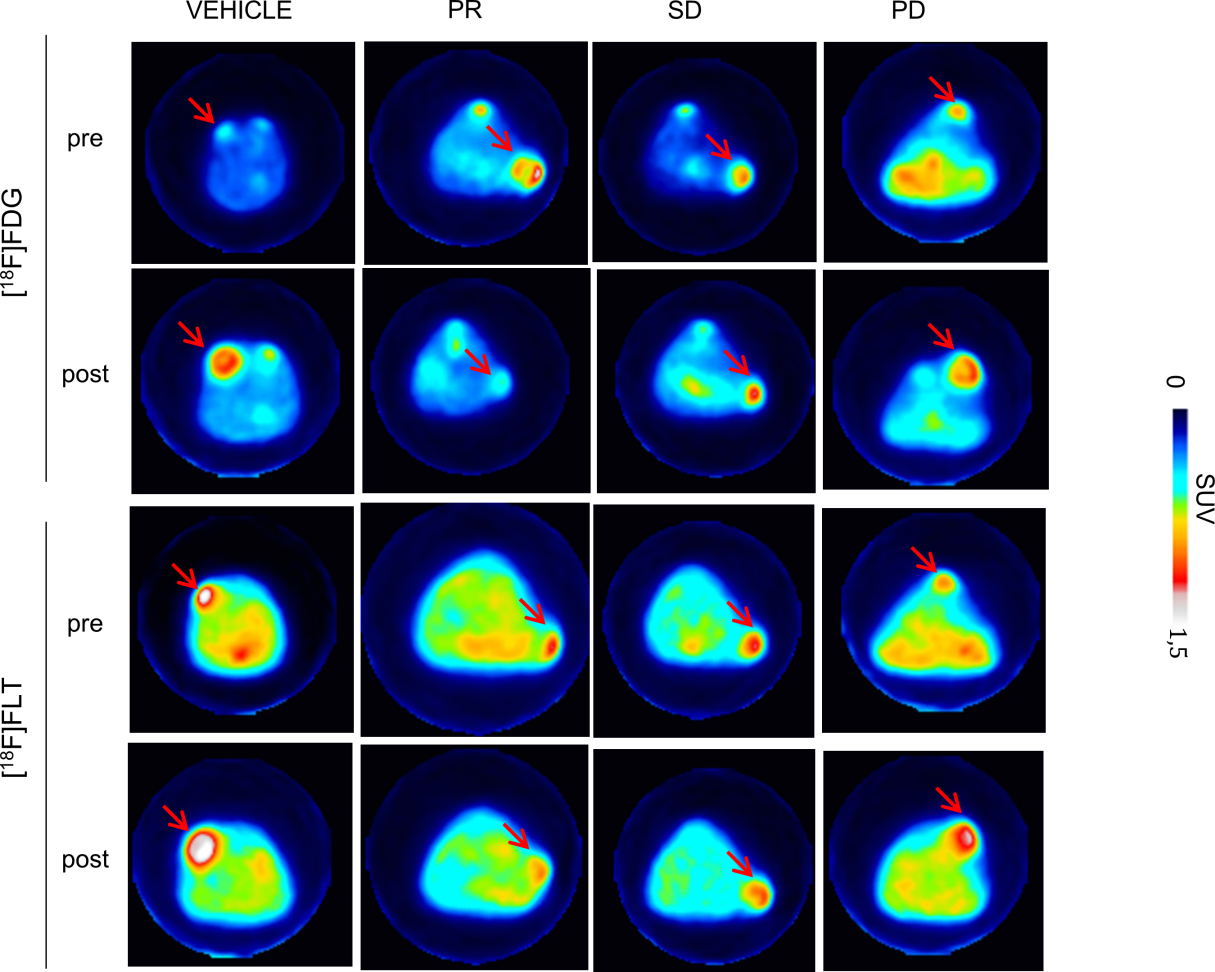
PET imaging of TNBC mouse model. Images of [^18^F]FDG and [^18^F]FLT scans of representative MDA-MB-468 xenografts mice performed pre and post PTX treatment. [^18^F]FDG and [^18^F]FLT uptake decreased in PR and SD, in contrast to the observed increase in PD and vehicle. Red arrows indicate cancer lesions. Color scale represents SUV value. PR = partial responder; SD = stable disease; PD = progressive disease.

**Fig 4:**
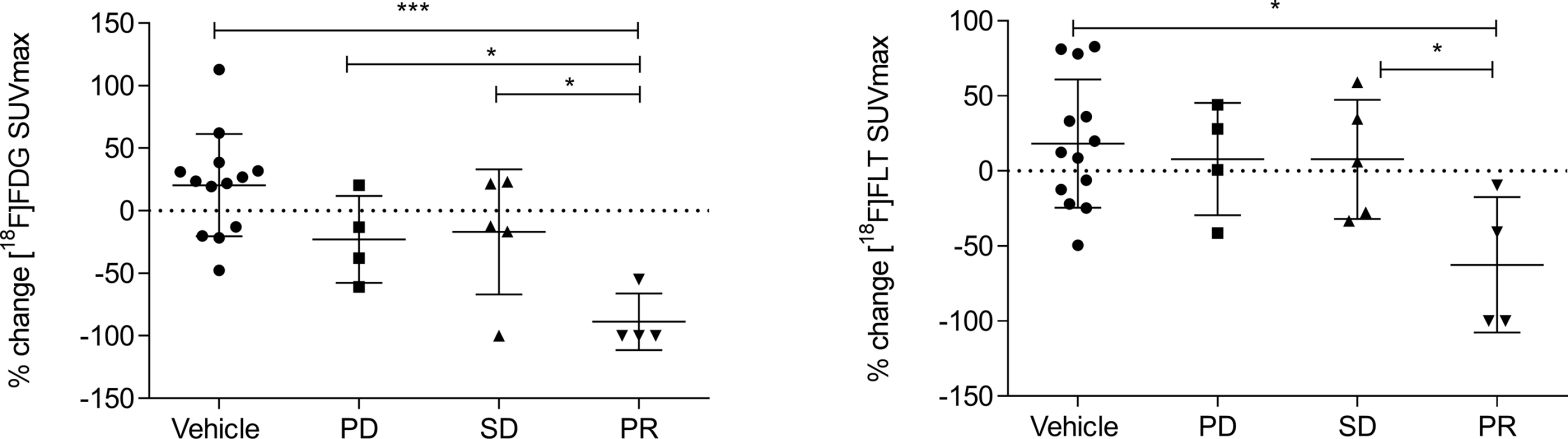
[^18^F]FDG and [^18^F]FLT uptake variations after treatment with PTX. [^18^F]FDG and [^18^F]FLT uptake expressed as percent variation (% change) in SUV_max_ (ΔSUV_max_) between baseline and post-therapy in vehicle and treated mice categorized on the basis of TVR. One-way ANOVA multiple comparison, *p < 0.05, **p < 0.01 and ***p < 0.001.

PTX treatment determined a reduction of both [^18^F]FDG and [^18^F]FLT uptake, which were found to be only slight for SD and more marked for PR, as shown in PET images (Fig 3). Treatment similarly affected [^18^F]FDG and [^18^F]FLT uptake in PR (S1 Fig). Moreover, PR uptake post treatment resulted significantly different from PD for [^18^F]FDG (p = 0.029). Percentage variation of the SUV_max_ (ΔSUV_max_) between baseline and post-therapy was more strongly associated with pathology outcome than with the absolute values. While [^18^F]FDG SUV_max_ reduction (ΔSUV_max_) from baseline to post-therapy was significant in PR (-88.69% ± 22.6%, p = 0.019), no significant modifications were observed in SD tumors (-16.89% ± 50.0%), where treatment caused only slight decreases of [^18^F]FDG SUV_max_, if any at all. In PD tumors [^18^F]FDG ΔSUV_max_ resulted highly heterogeneous (-22.83% ± 34.8%), which could be in part related to the presence of necrotic areas. [^18^F]FLT ΔSUV_max_ displayed a similar trend than that of [^18^F]FDG, with a significant reduction in PR (-62.56% ± 45.1%, p = 0.039), a stable trend in SD (7.74% ± 39.7%) and variable but not significant changes in PD (+7.91% ± 37.4%). FDG SUV_max_ variations appeared significantly different between partial responders and non-responders, that included both stable and progressive disease with statistical significance (p = 0.003). In detail, [^18^F]FDG SUV_max_ decrease in PR was significantly higher than that of vehicle (p = 0.0001, Fig 4) and that of PD and SD considered alone (p = 0.024 and p = 0.030 respectively, Fig 4) while [^18^F]FLT SUV_max_ decrease in PR was significantly higher only than that of vehicle and SD (p = 0.026 and p = 0.049 respectively, Fig 4). PTX treatment induced also a comparable reduction, although not significant, of both MTV and TLG or TLP, indicating that [^18^F]FDG and [^18^F]FLT distribution were similarly modulated by PTX (S2 Fig). The high heterogeneity in radiotracers volume distribution observed in PD mice could result from the presence of necrotic regions within large tumors.

### SUV_max_ variations represent a better parameter to evaluate response to therapy

**Fig 5:**
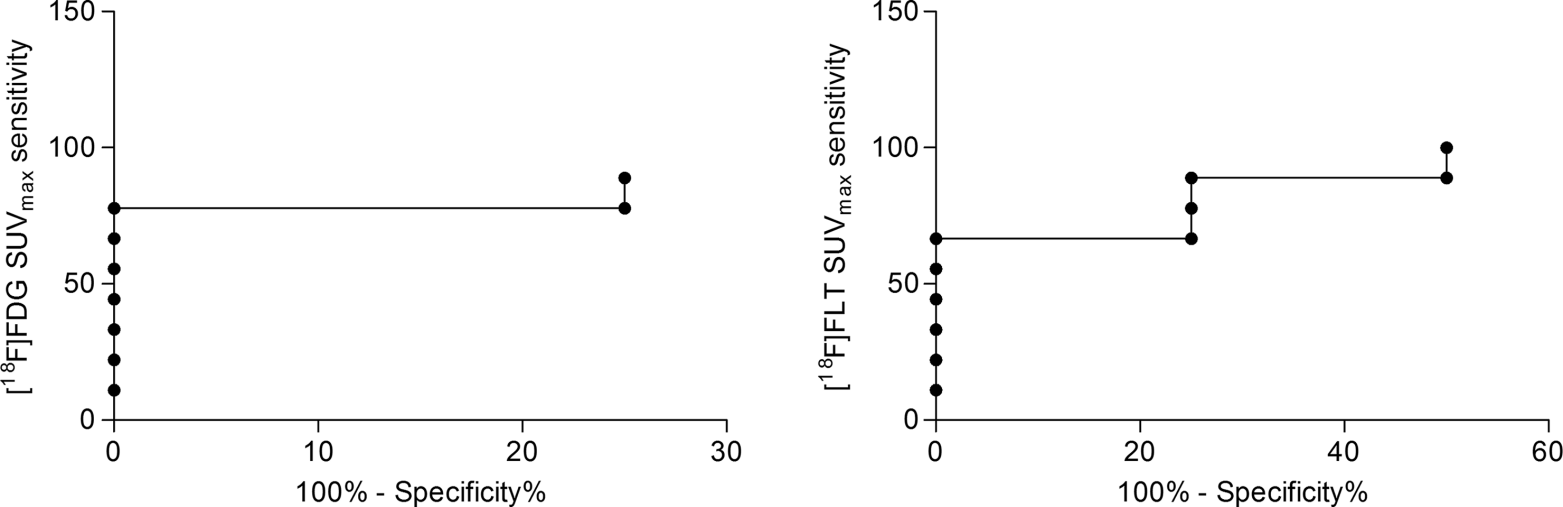
ROC curve of ΔSUV_max_ to predict MDA-MB-468 response. ROC analysis of [^18^F]FDG and [^18^F]FLT ΔSUV_max_ for prediction of different response to PTX therapy in the TNBC model. Optimal cut-off point was defined for [^18^F]FDG as -80.4% (89% sensitivity; 75% specificity) and for [^18^F]FLT as -70.7% (100% sensitivity; 50% specificity).

Our data indicated that variations of [^18^F]FDG SUV_max_ offered a better accuracy in defining response to NAC with PTX and in differentiating pathological partial responders from non-responders. The area under the curve (AUC) of ROC curves for [^18^F]FDG and [^18^F]FLT ΔSUV_max_ revealed a similar performance, distinguishing between responding and non-responding lesions, as classified by the TVR criteria with an accuracy slightly higher for [^18^F]FDG (AUC = 0.903, p = 0.025, and AUC = 0.889, p = 0.031 for [^18^F]FDG and [^18^F]FLT, respectively) (Fig 5). According to ROC analysis, a cut-off value of -80.4% offered for [^18^F]FDG ΔSUV_max_ the best accuracy in predicting non-responder lesions, with a sensitivity and specificity of 89% and 75%, respectively. ΔSUV_max_ for [^18^F]FLT was also an accurate prognostic factor leading to an optimal cut-off value of -70.7%, (100% sensitivity and 50% specificity), but resulted inferior to [^18^F]FDG.

TLG and TLP, as well as [^18^F]FDG and [^18^F]FLT MTV, showed a smaller accuracy in distinguishing partial responders from SD or PD compared to ΔSUV_max_ and they did not provide a clear cut-off (data not shown).

## Discussion

The objective of this study was to evaluate PET as an accurate tool to discriminate TNBC treatments’ responders. With this purpose, we used SCID mice bearing human MDA-MB-468 lesions of different size and, after classification of responders using an adapted RECIST criteria based on volumetric measurement of tumors, we evaluated response to PTX treatment comparing in the same set of mice [^18^F]FDG and [^18^F]FLT PET. Several breast cancer cell lines are currently used to study triple negative tumours; we took advantage of MDA-MB-468 which has been identified as ER-, PR- and HER- basal breast cancer cell, as approximately the 80% of TNBC [29]. Moreover, MDA-MB-468 cells display high Ki67 and EGFR expression and form cohesive grape-like or stellate structures consistent with the more invasive phenotype characterizing the TNBC human situation [30,31].

Tumor response to PTX treatment appeared variable in our study, revealing a high heterogeneity of volume variations, which was independent from the initial lesion size. The histopathological characteristic of MDA-MB-468 tumor and its typical microenvironment might act on PTX distribution and efficacy. Indeed, the presence of poor vascularized sub regions within the tumor although mimicking the clinical situation might influence PTX response [32].

Although TNBC represents an invasive and highly aggressive subtype of BC, using pCR as a surrogate endpoint, there are evidences that TNBC is a chemo-responsive disease [4]. However, while patients with TNBC responding to NAC have a relatively good prognosis, those without response display an extremely poor outcome, with a higher risk of relapse [4]. Hence, the possibility to evaluate the early efficacy of NAC is of fundamental importance for the clinical management of patients, tailoring the best treatment option on the basis of the initial response. NAC for TNBC, which is usually performed with a combination of taxanes and anthracyclines, has been performed with taxanes alone to focus the study in understanding changes in glucose metabolism and proliferation as potential markers of TNBC responsiveness. Indeed, [^18^F]FDG and [^18^F]FLT PET have been used to evaluate changes in glucose metabolism and proliferation triggered by treatment in our model of TNBC, which has been known to not show an inflammatory phenotype that could produce a bias in the interpretation of the results obtained using [^18^F]FDG.

Many studies have been performed to investigate the use of [^18^F]FLT as biomarker of response to treatment in comparison to [^18^F]FDG in preclinical models of cancer. The superiority of [^18^F]FDG compared to [^18^F]FLT or *viceversa* has been clearly demonstrated to be dependent on both the cell lines and treatment type and on the study design [16,17,18,19,20,21,22]. In the A2780 ovarian cancer model [^18^F]FDG and [^18^F]FLT were compared in different animals and their diagnostic efficacy evaluated at baseline and at different times after the beginning of treatment. [^18^F]FDG and [^18^F]FLT displayed a different behavior in response to Paclitaxel plus Carboplatin [22]. Animals bearing HCT116 tumors were used to evaluate the ability of [^18^F]FDG or [^18^F]FLT to assess the effect of Docetaxel alone or with the kinase inhibitor Selumetinib. No modifications in [^18^F]FDG uptake and a significant increase in [^18^F]FLT 7 days after treatment were observed [20]. Both [^18^F]FDG and [^18^F]FLT were used to identify the effect of PTX conjugated to RGD peptide in the MDA-MB-435 TNBC model. Although it is still unclear whether MDA-MB-435 would represent a model of triple negative breast cancer or a melanoma [30,31], the effect of RGD-PTX seemed to be not significantly related neither to [^18^F]FDG nor to [^18^F]FLT [21]. In our TNBC model PTX treatment clearly demonstrated an effect on proliferation as depicted by the significant correlation between tumor reduction and Ki67 reduction. Nevertheless, MDA-MB-468 bearing mice performing PET imaging displayed that [^18^F]FLT variations were not more indicative than [^18^F]FDG SUV_max_ variations in defining response to therapy.

Only few data are available to support the use of [^18^F]FLT as a marker of TNBC response to NAC, although it can provide higher specificity, since its accumulation in inflammatory areas is less significant than [^18^F]FDG [33]. It has been demonstrated that [^18^F]FLT PET is able to detect therapy-induced proliferation changes as early as 1 week after FEC (5-fluorouracil, epirubicin, cyclophosphamide) chemotherapy, discriminating between responders and SD patients with stage I-IV breast cancer [34]. In another study, the predictive value of changes in [^18^F]FLT SUV after the first cycle of chemotherapy was demonstrated in patients with metastatic breast cancer [35]. Monitoring response to NAC therapy is of great importance since it allows the early switch for alternative treatment. Moreover, in a small population of locally advanced BC patients, Crippa et al. demonstrated the good sensitivity, specificity and AUC of [^18^F]FLT PET for early monitoring of response after a single cycle of NAC [36]. On the other hand, in a heterogeneous population of primary BC patients, [^18^F]FLT revealed only a marginal predictive value of therapeutic response after one cycle of NAC, displaying a good AUC despite highly variable chemotherapy regimens [37]. In a similar way, Woolf reported a reduction of [^18^F]FLT SUV_max_ after a single cycle of NAC, but they demonstrated that neither the baseline value, nor the variation of SUV_max_ after therapy was able to predict treatment response in an heterogeneous population of primary BC [38]. Recent studies report that TNBC tumors usually have higher metabolic activity than those of other phenotypes, being aggressive and [^18^F]FDG avid [39]. Indeed, [^18^F]FDG uptake has been largely used to efficiently distinguish TNBC patients responsive to different NAC therapies, compared to other subtypes such as HER-2 positive tumors [40,41,42]. The nature of PET images (i.e., low spatial resolution, high statistical uncertainty noise, and ill-defined margins) renders the MTV and TLG/TLP quantification particularly hard. Moreover, the inaccuracy of visual delineation of tumor is subjected to both intra and inter-operator variability. In order to avoid these limitations, we took advantage from a graph-based algorithm for MTV delineation [26] which differs from conventional approaches since it is more accurate in noisy and low contrast images. Evaluation of tumour response to PTX has been made according to the RECIST criteria adapted to the use of volumetric measurements and it has been defined as tumor volume response (TVR), considering the reduction of tumour volume instead of that of longest diameter [23,43]. Relying on the categorization of responders using TVR evaluation, we observed a general and similar decrease of both [^18^F]FDG and [^18^F]FLT in PR whereas a stable and heterogeneous trend was found in SD and PD. [^18^F]FDG changes in SUV_max_ offer the best cut-off value to differentiate responders, indicating a [^18^F]FDG SUV_max_ decrease of 80.4% with high specificity (89%) and sensitivity (75%). Moreover, [^18^F]FLT SUV_max_ variations from baseline to post-therapy appeared more heterogeneous than glycolysis variations, detected by [^18^F]FDG, leading us to fail in demonstrating a better usefulness of [^18^F]FLT as biomarker of TNBC response to NAC. Nevertheless, our data seem to be in line with other previous reports [38], suggesting the main benefit of [^18^F]FLT PET as an imaging biomarker of proliferation pre-chemotherapy, rather than a biomarker of tumor response to therapy in TNBC.

Many studies apply ROC curves to define an optimal threshold value of radiotracers uptake, able to discriminate responders [14,36,41,42]. The differences in the published threshold value are caused by several factors, which have to be taken into account, such as the definition of good pCR, the time of PET, or the chemotherapy regimen. It has been noted that the use of different therapeutic agents may affect [^18^F]FLT uptake regardless the direct effect on proliferation, depending on their influence on nucleoside metabolism and on cell cycle [22].

The use of Paclitaxel on a chemotherapeutic regimen has been shown to have minimal effect on [^18^F]FLT uptake, since it induces cell cycle arrest in an advanced point which doesn’t affect TK1 expression or change cell proliferation, even though it reduces tumor growth [22]. Moreover, it has to be considered that other mechanisms, including the use of salvage or de novo pathway for DNA synthesis, could influence [^18^F]FLT uptake [14].

## Conclusions

In conclusion, many works have been performed investigating the role of [^18^F]FDG or [^18^F]FLT in the assessment of tumor response to therapy in TNBC, producing heterogeneous results without a clear indication in favour of the usefulness of [^18^F]FDG rather than [^18^F]FLT or *viceversa*. Although many studies have been performed to assess the utility of [18F]FDG and [18F]FLT as markers of tumor response to treatments, our study represents the first head to head comparison between these two tracers in the same subjects in TNBC. Out data demonstrated the comparable sensitivity of [^18^F]FDG and [^18^F]FLT SUVmax in the evaluation of responders based on Tumor Volume Response. TNBC models obtained from patients samples (Patient Derived Xenografts) may better mimic the heterogeneity of the disease and they will be used to validate the study.

## Supporting information

S1 Fig [^18^F]FDG and [^18^F]FLT SUVmax. [^18^F]FDG and [^18^F]FLT SUVmax calculated at the baseline (pre) and post treatment (post) in vehicle and treated mice categorized on the basis of TVR. Multiple comparison assessed using 2-way ANOVA analysis and *p < 0.05, **p < 0.01 and ***p < 0.001.
(TIF)

S2 Fig Variation of [^18^F]FDG and [^18^F]FLT uptake. [^18^F]FDG and [^18^F]FLT uptake expressed as percent variation (% change) in A) TLG (Total Lesion Glycolysis) or TLP (Total lesion Proliferation) and B) MTV (Metabolic Tumor Volume) between baseline and post-therapy in vehicle and treated mice categorized on the basis of TVR.
(TIF)

S3 Fig ARRIVE guidelines checklist. (DOCX)
