# Supplementary figures and images for: [18F]FDG and [18F]FLT PET for the evaluation of response to neo-adjuvant chemotherapy in a model of triple negative breast cancer
Source: PLoS One. 2018 May 23;13(5):e0197754. doi: 10.1371/journal.pone.0197754 (PMC5965848; doi:10.1371/journal.pone.0197754)

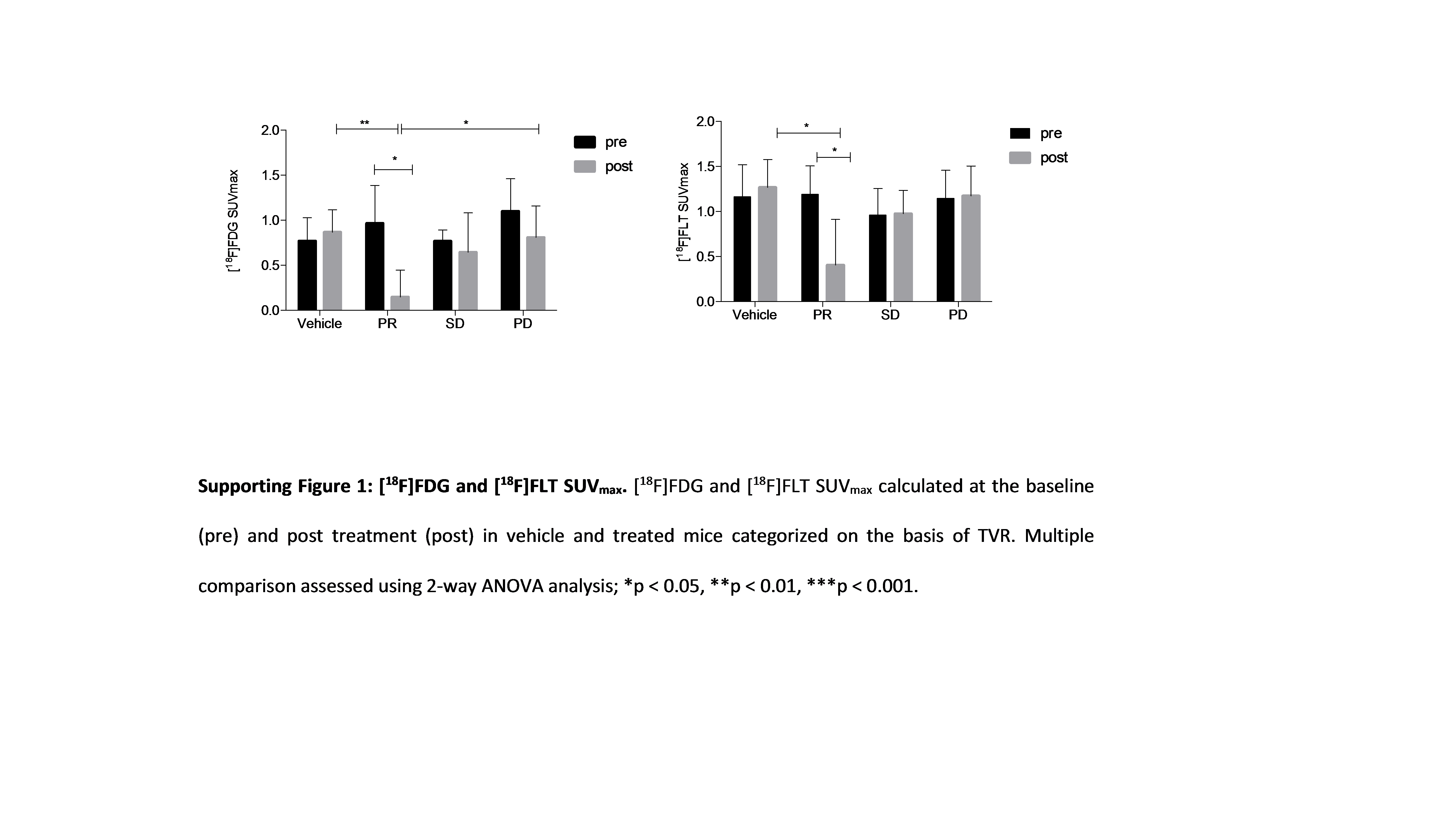

Supplement: S1 Fig — [18F]FDG and [18F]FLT SUVmax calculated at the baseline (pre) and post treatment (post) in vehicle and treated mice categorized on the basis of TVR. Multiple comparison assessed using 2-way ANOVA analysis and *p < 0.05, **p < 0.01 and ***p < 0.001. (TIF) [file pone.0197754.s001.tif]

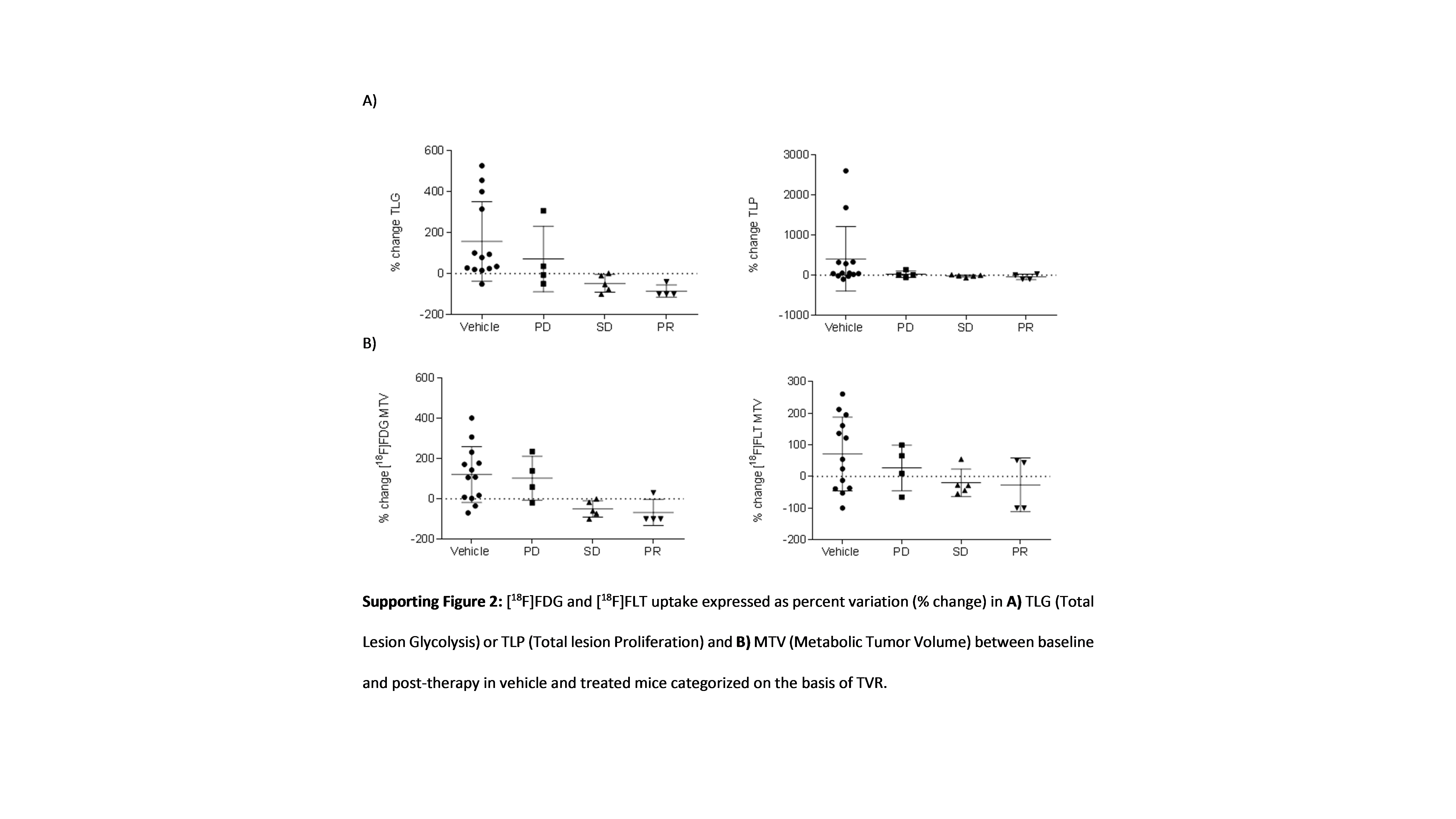

Supplement: S2 Fig — [18F]FDG and [18F]FLT uptake expressed as percent variation (% change) in A) TLG (Total Lesion Glycolysis) or TLP (Total lesion Proliferation) and B) MTV (Metabolic Tumor Volume) between baseline and post-therapy in vehicle and treated mice categorized on the basis of TVR. (TIF) [file pone.0197754.s002.tif]
