# Supplementary material for: [18F]FDG and [18F]FLT PET for the evaluation of response to neo-adjuvant chemotherapy in a model of triple negative breast cancer
Source: PLoS One. 2018 May 23;13(5):e0197754. doi: 10.1371/journal.pone.0197754 (PMC5965848; doi:10.1371/journal.pone.0197754)

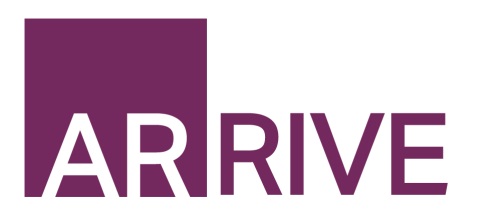
**Supporting Figure 3 ARRIVE Guidelines Checklist.**

The ARRIVE Guidelines Checklist

Animal Research: Reporting In Vivo Experiments

Carol Kilkenny^1^, William J Browne^2^, Innes C Cuthill^3^, Michael Emerson^4^ and Douglas G Altman^5^

*^1^The National Centre for the Replacement, Refinement and Reduction of Animals in Research, London, UK, ^2^School of Veterinary Science, University of Bristol, Bristol, UK, ^3^School of Biological Sciences, University of Bristol, Bristol, UK, ^4^National Heart and Lung Institute, Imperial College London, UK, ^5^Centre for Statistics in Medicine, University of Oxford, Oxford, UK.*

|  | | ITEM | RECOMMENDATION | Section/ Paragraph |
| --- | --- | --- | --- | --- |
| 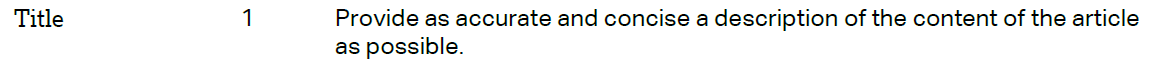 | | | Title (p 1) |  |
| 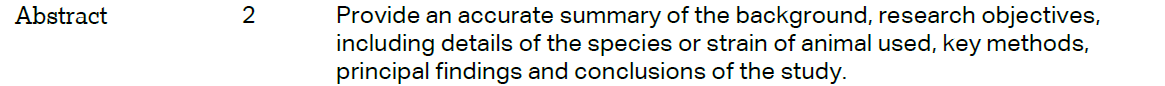 | | | Abstract (p 2) |  |
| INTRODUCTION | | |  |  |
| 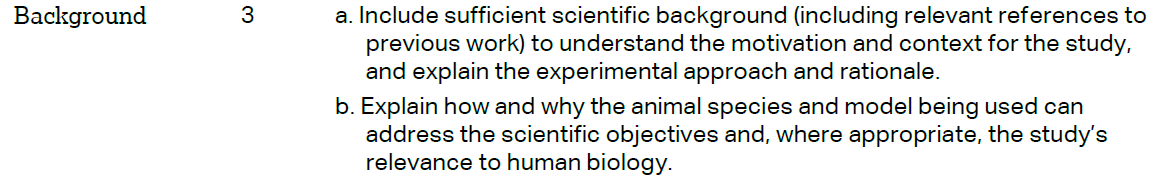 | | | Introduction  (p 3-4) |  |
| 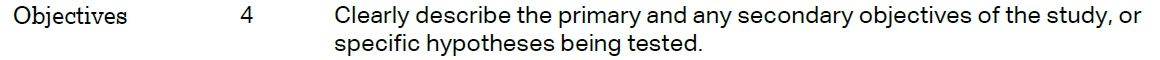 | | | Introduction (p 4) |  |
| METHODS | | |  |  |
| 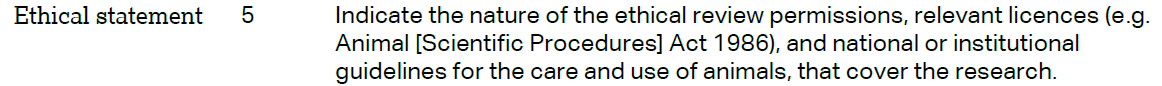 | | | Material and methods/ Animal experiments (p 5) |  |
| 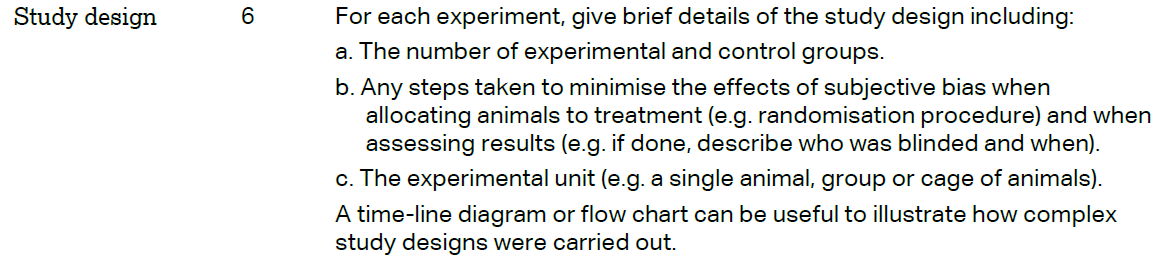 | | | Material and methods/ Treatment protocol (p 5-6) |  |
| 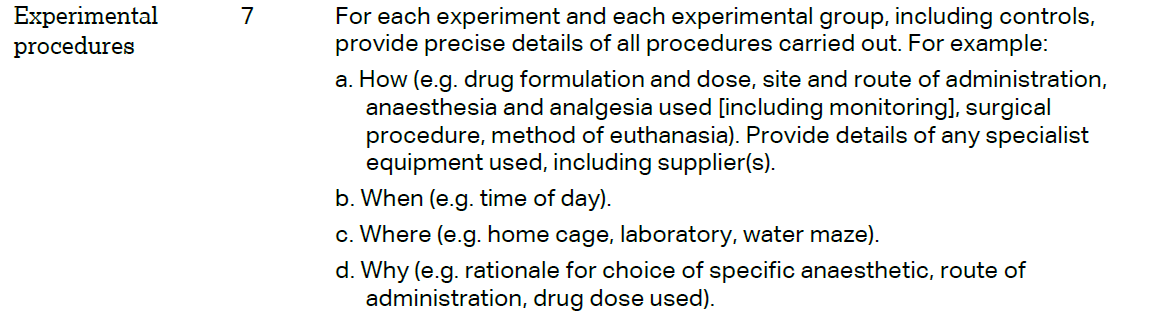 | | | Material and methods/ Treatment protocol (p 5-6) |  |
| 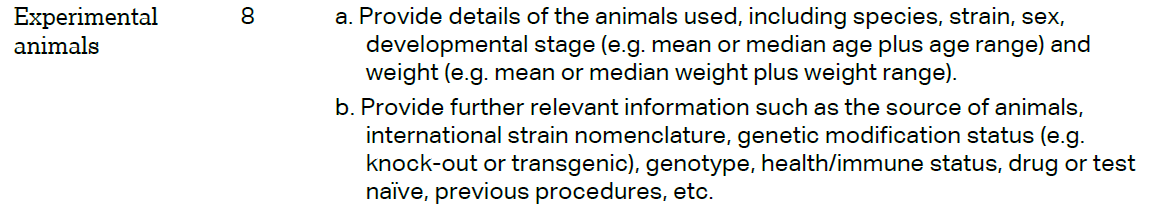 | | | Material and methods/ Animal experiments (p 5) |  |

The ARRIVE guidelines. Originally published in *PLoS Biology*, June 2010^1^

| 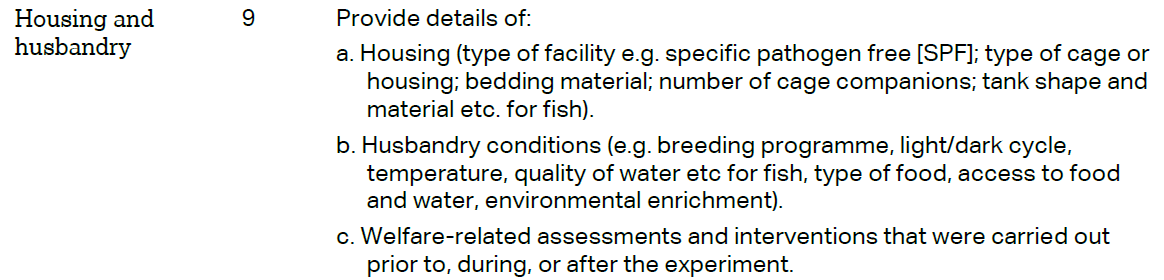 | Material and methods/ Animal experiments (p 5) | |
| --- | --- | --- |
| 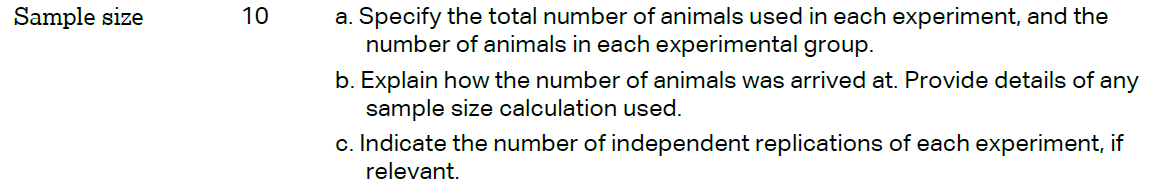 | Material and methods/ Treatment protocol (p 5-6) | |
| 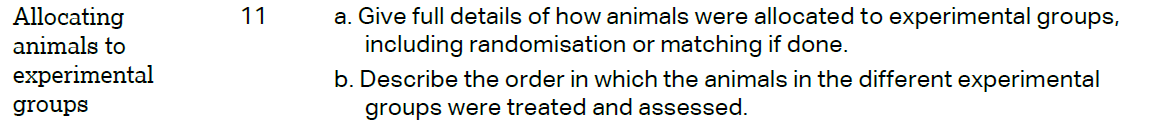 | Material and methods/ Treatment protocol (p 5-6) | |
| 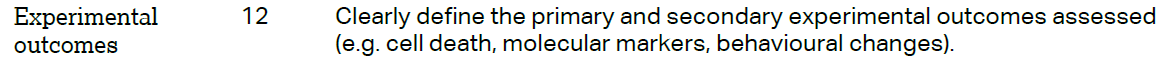 | Material and methods (p 5-7) | |
| 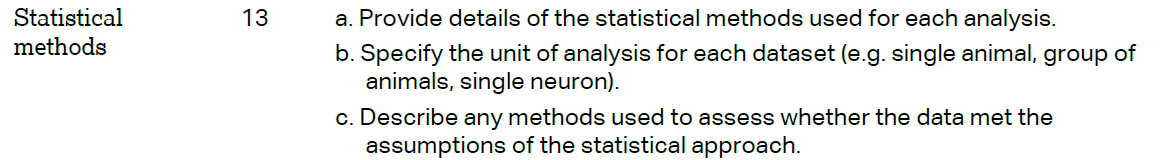 | Material and methods/ Statistical analysis (p 7) | |
| RESULTS |  | |
| 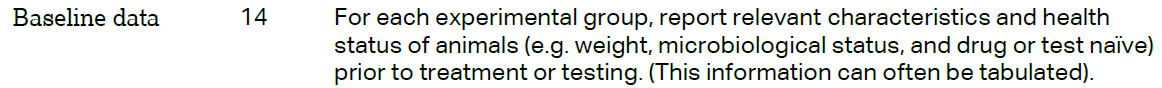 | Results/ Tumor weight after treatment correlates with Ki67 expression | |
| 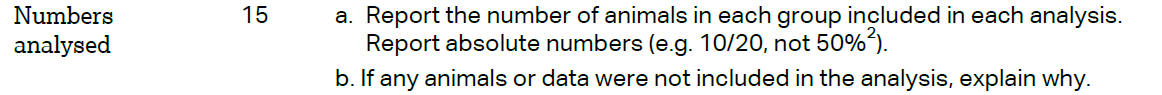 | Results/ Table 1 | |
| 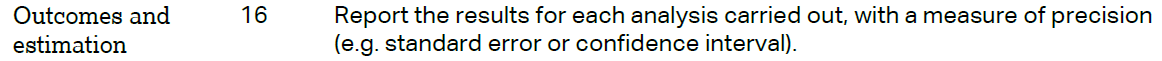 | Results | |
| 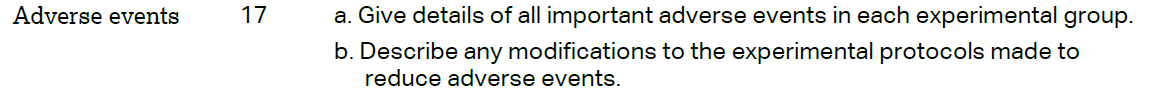 | Results/ Tumor weight after treatment correlates with Ki67 expression | |
| DISCUSSION |  | |
| 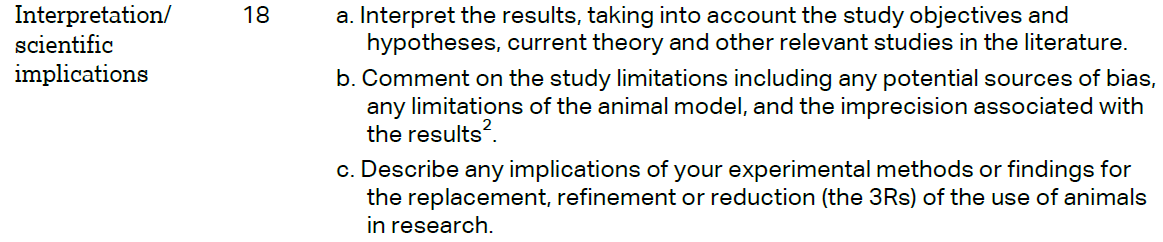 | Discussion | |
| 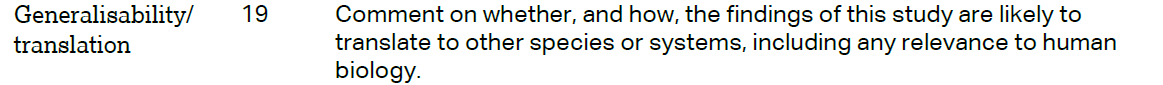 | Discussion | |
| 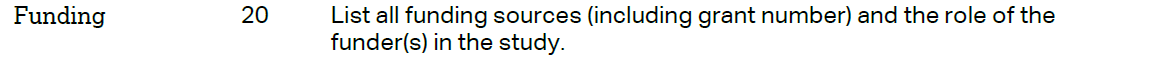 | | Acknowledgments |


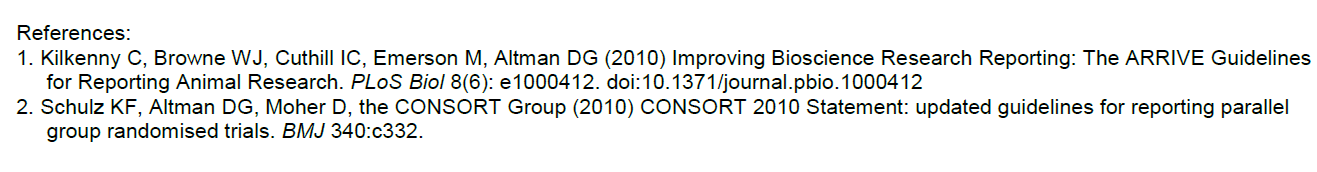

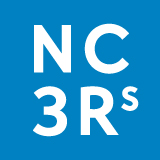

Supplement: S3 Fig — (DOCX) [file pone.0197754.s003.docx]
